# Effect of natural silk fibers and synthetic fiber-reinforced composites on the cytotoxicity of fibroblast cell lines

**DOI:** 10.34172/joddd.40900

**Published:** 2024-06-24

**Authors:** Mutiara Annisa, Dyah Irnawati, Widowati Siswomihardjo, Siti Sunarintyas

**Affiliations:** Department of Dental Biomaterials, Faculty of Dentistry, Universitas Gadjah Mada, Yogyakarta,Indonesia

**Keywords:** Bombyx mori, Cytotoxicity, Fiber-reinforced composites, Fibroblast cell lines, Samia ricini

## Abstract

**Background.:**

Synthetic fibers have many benefits in clinical practice; however, they cause microplastic pollution, and their unaffordable price increases treatment costs. Natural silk fibers require biocompatibility assessment. This study investigated the effects of natural and synthetic fiber-reinforced composites (FRCs) on the cytotoxicity of fibroblast cell lines.

**Methods.:**

Three commercial synthetic fibers (polyethylene, quartz, and E-glass) and two silk fibers from *Bombyx mori* and *Samia ricini* cocoons were employed. These fibers were made into FRC samples (n=6) by impregnation in flowable composite using a brass mold (25×2×2 mm). NIH/3T3 mouse fibroblasts were cultured in Dulbecco’s modified eagle medium, supplemented, and seeded in 2×10^4^ cells/mL. They were stored at 37 °C under 5% CO_2_ for 24 hours. The FRC samples were made into powder, eluted in dimethylsulfoxide, continued with PBS, supplemented with Dulbecco’s modified eagle medium (DMEM), and exposed to cells for 24 hours. Blank (medium only) and control (cells and medium) samples were included. Subsequently, MTT was added for 4 h and read by enzyme-linked immunosorbent assay (λ=570 nm). Cell viability (%) was calculated and analyzed using one-way ANOVA (α=0.05).

**Results.:**

All groups of FRCs showed>80% cell viability. One-way ANOVA showed no significant difference between FRC groups regarding the viability of fibroblast cell lines (*P*>0.05).

**Conclusion.:**

Both natural silk and synthetic fibers exhibit low cytotoxicity to fibroblast cell lines. *B. mori* and *S. ricini* silk fibers showed the potential to be used as alternative synthetic fibers.

## Introduction

 Fibers have been widely used in dental clinical settings as fiber-reinforced composites (FRCs), mostly for applications such as dental restorations, periodontal splints, endodontic posts, orthodontic retainers, fixed partial dentures, and removable dentures.^1^ The use of FRCs relies on their exceptional properties, such as physicomechanical, bonding, and viscoelastic capabilities and biocompatibility, which are set forth by the specific fiber and matrix used.^1,2^ In addition, using FRCs in clinical practice enables minimal preparation of the neighboring tooth structure.^2^

 Dental composites are strengthened using various fibers, including synthetic materials such as polyethylene, carbon, glass, Kevlar (p-phenylene diamine), and plant- and animal-derived natural fibers. These fibers can be arranged in unidirectional, braided, and woven patterns.^1,3^ Although synthetic fibers offer benefits such as durability, high tensile strength, elastic modulus, and ultimate strain in FRCs, their use is often expensive, leading to high dental treatment costs.^4,5^ Furthermore, this synthetic material is nonbiodegradable, is not eco-friendly, and contributes to microplastic contamination. To address these limitations, interest is growing in natural fibers as substitutes for synthetic fibers. These natural fibers can be cellulose-rich plant or animal fibers mainly made up of proteins.^6^ Silk fiber is considered a potential animal-derived fiber because its mechanical qualities are superior to those of plant fibers, and its specific mechanical performance is comparable to those of glass and polyethylene fibers.^5,7^ The silk fiber that can be used originated from *Bombyx mori*, a controlled silkworm fed with mulberry. In a study by Shah et al,^8^ the tensile strength and specific strength of a composite laminate made from *B. mori* silk fiber and glass fiber were comparable. This finding is consistent with those reported by Sunarintyas et al,^5^ who used an identical silk fiber material and demonstrated a flexural strength similar to that of polyethylene FRCs. Despite limited evidence, the potential use of the non-mulberry cocoon type is exemplified by *Attacus atlas*, *Cricula trifenestrata*, and *Samia ricini* in terms of the quality of the silk fiber and its protein components, such as sericin and fibroin.^7,9,10^

 However, the biocompatibility issue regarding the effect of FRC use on biological tissue is widely discussed because FRCs have the same resin matrix as conventional resin composites, such as methyl methacrylate (MMA), which may cause allergy, contact dermatitis, and mucous membrane irritation.^11,12^ Incorporating fiber and resin matrix also contributes to chemical reactions that affect cytocompatibility.^6,10^ ISO 10993-1 suggests appropriate steps for the preliminary assessment of biological compatibility of medical devices through in vitro evaluation of cytotoxicity.^10,13^ Thus, this study aimed to investigate the effects of natural and synthetic FRCs on the cytotoxicity of fibroblast cell lines.

## Methods

 This experimental laboratory study received ethical clearance from the Ethics and Advocacy Commission of the Faculty of Dentistry at Universitas Gadjah Mada (Approval No. 73/UN1/KEP/FKG-RSGM/EC/2023). The NIH/3T3 mouse fibroblast cell line (ATCC, Old Town, MD, USA) was used in the cytotoxicity assay. Cell culture was performed using Dulbecco’s modified eagle medium (DMEM; Invitrogen, Carlsbad, CA, USA). The supplementation included fetal bovine serum (FBS, Sigma-Aldrich, USA), penicillin (Gibco, Grand Island, NY, USA), and gentamicin (Gibco). Five types of fibers consisting of two types of natural silk fibers and three types of synthetic fibers were employed. The details of the materials used for specimen preparation are provided in Table 1.

**Table 1:** Information on the materials used for specimen making in this study

**Materials**	**Manufacturer**	**Description**	**Number of specimens**
*Bombyx mori *silk fiber	Local silk from Wajo, South Sulawesi, Indonesia	Domesticated silk fiber derived from the cocoon of *Bombyx mori, *tailored into silk ribbon in unidirectional configuration	6
*Samia ricini*silk fiber	Local silk from Kulon Progo, Yogyakarta, Indonesia	Wild silk fiber derived from the cocoon of *Samia ricini, *tailored into silk ribbon in unidirectional configuration	6
Polyethylene fiber	Kerr Construct, USA	Commercially available synthetic fiber made of UHMWPE in woven configuration	6
Quartz fiber	Quartz Splint, France	Commercially available synthetic fiber made of quartz in unidirectional configuration	6
E-glass fiber	everStick^TM^, Stick Tech Ltd, Finland	Commercially available synthetic fiber made of E-glass impregnated with bis-GMA and PMMA in unidirectional configuration	6
Flowable composite resin	DenFil Flow, Vericom, Korea	Light-cured flowable composite resin used to make specimens of FRCs	-

**Figure 1:**
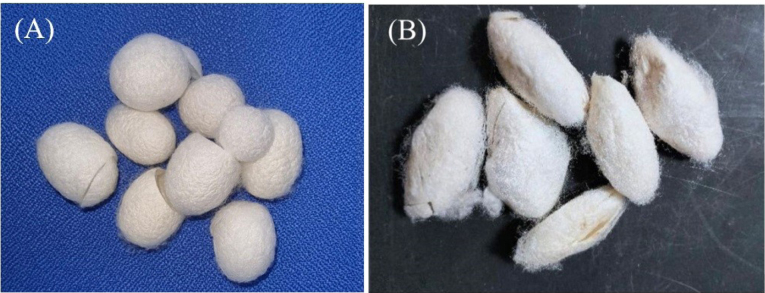
Cocoon of silkworm. (A) *Bombyx mori *and(B) *Samia ricini*

Table 1 presents the specifications of the natural and synthetic fibers used in this study. While synthetic fibers are readily available, both natural silk fibers must be tailored before they can be transformed into FRC specimens. Initially, the cocoons of *B. mori* and *S. ricini* (Figure 1) were subjected to a degumming technique, as described by Rameshbabu et al,^14^ to remove sericin. The silk fibers were then extracted and twisted into threads.

###  Preparation of the silk ribbon 

 The unidirectional silk ribbons from each silk fiber were prepared following the protocol of Sunarintyas et al.^5^ Silk fibers of *B. mori *and *S. ricini*were weighed to 0.155 g each and then arranged in a brass mold (80 × 2 mm), followed by impregnation with flowable composite resin. The resulting silk ribbon was stored at 4 °C.

###  Specimen preparation

**Figure 2:**
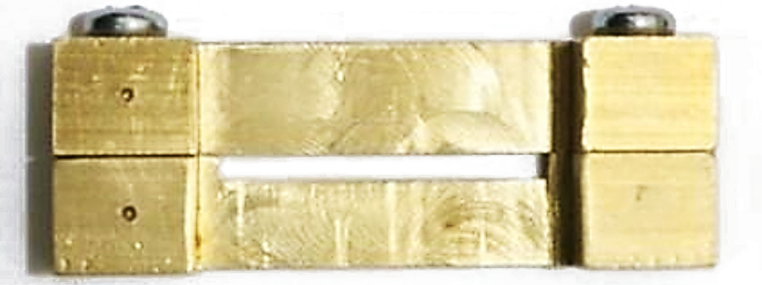
The sample preparation process

 The specimens were prepared according to the procedure outlined by Frese et al,^15^ with a few modifications. Aseptically, a brass mold measuring 25 × 2 × 2 mm was positioned and secured onto a microscope slide. A layer of flowable composite resin was applied to the lower portion of the mold, which occupied roughly one-third of its height. Subsequently, each fiber was inserted into the mold and saturated with the composite resin. After that, another layer of composite resin was added to fill the mold. Subsequently, they were delicately compressed using a different microscope slide and subjected to light curing following the manufacturer’s guidelines. Figure 2 is a schematic representation of the sample preparation process.

###  Cell culture

 Cell culture was performed according to the method employed by Klein-Junior et al.^16^ NIH/3T3 murine fibroblasts were regularly cultured in DMEM. The solution was enriched with 10% fetal bovine serum, 100 U/mL penicillin, 100 U/mL streptomycin, and 100 μg/mL gentamicin. The samples were arranged in 96-well plates, and the cells were distributed onto the samples at a concentration of 2 × 10^4^ cells/mL. The cells were subsequently placed in a humidified incubator at 37°C with a carbon dioxide concentration of 5% for 24 hours.

###  Preparation of the stock solution 

 Before the cytotoxicity test, all FRC specimens were stored in a sterile saline solution for 24 hours at room temperature. Each group of FRCs had six specimens (n = 6). Subsequently, the fibers were pulverized and dissolved in dimethylsulfoxide, further diluted with PBS, and supplemented with DMEM to achieve a stock solution concentration of 100 µg/mL for each fiber type. The stock solutions were filtered using a 0.22-µm Millipore membrane (Millipore; Billerica, MA, USA) and stored at 4 °C.^12^

###  Cell viability

 The 3-(4,5-dimethylthiazol-2-yl) 2,5-diphenyl tetrazolium bromide (MTT) cytotoxicity test was performed according to ISO 10993-1. A fibroblast cell line consisting of 2 × 10^4^ cells/mL in supplemented DMEM was placed in 96-well plates and cultured for 24 hours until a semiconfluent monolayer was formed. Subsequently, they were subjected to several fiber stock solutions, with six replications for each group (n = 6). The medium without cells was used as the blank, whereas the medium with cells was utilized as the control. The incubation period for the treated and untreated groups lasted 24 hours at 37 °C with a CO_2_ concentration of 5%. Following a 24-hour exposure period, the MTT assay was performed by introducing MTT and allowing it to incubate. Following a 4-hour incubation period, formazan formation was assessed for each treatment concentration using an enzyme-linked immunosorbent assay reader set at 570 nm. The cell viability percentage in the treated cells was determined relative to that in the control cells using the following formula:


$$Cytoviability\;\%=\frac{OD\;treated-OD\;blank}{OD\;control-OD\;blank}$$

###  Statistical analysis

 Data were reported as the average percentage of cell viability ± standard deviation. The effect of different types of FRCs on the viability of fibroblasts was determined through statistical analysis using ANOVA.

## Results

 The cell viability of the fibroblast cell lines in the treated group was compared with that of the control cells (untreated group) (Table 2).

**Table 2:** Cell viability percentage of NIH/3T3 fibroblast cell line after 24 hours of exposure to various fiber-reinforced composites (FRCs)

**Groups**	**Cytoviability (%)±SD**
Fibroblast cell lines (control)	100.00 ± 35.47
*Bombyx mori *silk FRCs	100.44 ± 16.35
*Samia ricini*silk FRCs	114.47 ± 27.70
Polyethylene FRCs	103.07 ± 28.77
Quartz FRCs	102.41 ± 38.29
E-glass FRCs	118.20 ± 38.29

Table 2 indicates that all the categories of FRCs had cell viability percentages exceeding 80%, surpassing the percentage of the control group, which included only the medium and cells. Statistical analysis was extended using one-way ANOVA. The results (Table 3) indicated the lack of significant variations in the viability of the fibroblast cell line after 24 hours of exposure to all FRCs (*P* > 0.05).

**Table 3:** ANOVA summary of the cell viability of NIH/3T3 fibroblast cell line after 24 hours of exposure to various FRCs

	**Sum of square**	***df***	**Mean square**	**F**	***P*** ** value**
Between groups	1381.980	4	345.495	0.401	0.806
Within groups	21529.037	25	861.161		
Total	22911.017	29			

## Discussion

 Composites are artificial materials with multiple phases and a desirable combination of the most advantageous features from each phase.^17^ When polymers are used to reinforce composites, resulting in polymer matrix composites, they can be classified into two types: particle-reinforced types, such as dental composites, and fiber-reinforced types, such as dental FRCs. Fibers can be composed of various materials, including carbon, aramid, polyethylene, or glass, which fall into the categories of synthetic fibers. In addition, natural fibers are derived from plants, such as jute, coir, and sisal, and animal fibers, such as wool and silk.^18^ This material is designed to possess exceptional qualities for various therapeutic uses. However, potential changes and reactions that may occur in terms of biocompatibility must be considered.^19^

 This study examined the cytotoxic effects of synthetic and natural FRCs on a fibroblast cell line. Multiple in vitro test paradigms are available for assessing the cytotoxicity of dental biomaterials. The methods used include direct contact, where the materials come into direct contact with the cellular layer; indirect contact, where a barrier is placed between the cell and the biomaterial layer, such as agar overlay assay and filter diffusion; and extract method, where the extracts of materials are placed in contact with cells.^13^ An ideal in vitro test closely replicates the in vivo settings. However, certain factors must be considered, such as the nature of the biomaterial being examined (substances, solid, and powder) and the specific purpose of the test being undertaken. This study used the direct contact assay, which is the most sensitive method for assessing the cytotoxicity of medical devices. This assay can detect even weak cytotoxic effects caused by medical devices.^20,21^

 Cell lines are used based on their shape and uniform growth characteristics. Although primary cells have a less accurate representation of the oral environment than cell lines, the primary cells will differ in their developmental and cultural characteristics.^20^ Heravi et al^22^ used human gingival fibroblasts and cell lines to demonstrate a consistent cytotoxicity pattern throughout the experiment. They proposed that using a cell line is sufficient when comparing the cytotoxicity of different materials. Nevertheless, in the presence of a specific issue related to the dosage, primary cells are recommended. Therefore, using a fibroblast cell line in this investigation is justifiable.

 The MTT assay employed in this investigation is a straightforward, highly responsive, trustworthy enzymatic assay frequently used to evaluate the cytotoxicity of diverse medicinal and hazardous substances. The test relies on the metabolic activity of living cells to enzymatically convert a yellow, soluble tetrazolium salt (MTT) into a purple formazan dye.^23^ The MTT assay system is a superior and more precise test than the trypan blue exclusion assay because of its ability to quantitatively measure cell activity based on absorbance. This test allows for the accurate measurement of cell growth and death rates. The trypan blue test is a qualitative assay that alone determines cell viability.^23,24^

 The results of the present study demonstrated that the percentage of live cells exceeded 80%. This result indicates a noncytotoxic effect.^25^ Moreover, when subjected to one-way ANOVA analysis, the cell viability percentages of the commercially available synthetic and natural silk fibers were not significantly different (Table 3), suggesting that natural silk fibers can be considered equivalent to the already available fibers. Cytotoxicity is mainly caused by residual monomers resulting from the enhancement of the adhesion between the fiber and matrix rather than being solely attributed to the fiber. Nevertheless, using untreated fibers without supplementary silane or monomer treatment exhibited lower cytotoxicity.^26^ A possible explanation for the lack of toxicity to fibroblast cell lines by the currently used natural silk fibers, such as *B. mori* and *S. ricini*, could be attributed to this factor because neither additional silane nor treatment was performed. Furthermore, the presence of an interpenetrating polymer network structure may explain the low cytotoxicity of all fibers. This structure is formed because of the strong adhesion between the fiber and resin composite matrix. Consequently, the number of leftover monomers decreases, reducing toxicity.^27^

 Essentially, these two types of silk are made from the cocoons of *B. mori* and *S. ricini* silkworms. They consist of two central fibroin filaments joined by a layer of sericin.^28^ The domesticated mulberry silkworm *B. mori* exhibits a slight disparity in protein composition compared with the wild silkworm *S. ricini*. The primary structure of the wild silkworm consists of 100 repetitions of alternating poly-(L)-alanine (PA) and glycine domains. In contrast, *B. mori* primarily consists of glycine, alanine, and serine residues, with a greater abundance of glycine. The disparity between them influences their mechanical characteristics, although both possess the potential for cell adhesion and proliferation.^29^ Because of its high levels of hydrophilic, wet, and positively charged amino acids, *S. ricini* silk is expected to exhibit greater cell attachment and proliferation than mulberry silk.^30^ This accounts for the enhanced cell viability observed with *S. ricini* in this study. This study confirms previous findings that evaluated the biocompatibility of sericin-free silk fibers for ligament tissue engineering using in vitro and in vivo tests. The results indicate that the silk fibers exhibit minimal toxicity and demonstrate a sustained increase in biocompatibility on days 1, 2, and 3.^31^ Past and current investigations have used sericin-free silk fibers that have completed the degumming process because sericin can trigger a negative immunological response when implanted in the human body and can generate an inflammatory reaction.^28,31^

 The E-glass FRC had the highest cell viability percentage among the synthetic fibers tested. However, this difference was not statistically significant compared to other fibers, such as polyethylene and quartz, which had cell viability percentages of 118.20 ± 38.29%, 103.07 ± 28.77%, and 102.41 ± 38.29%, respectively. The present study aligns with previous research that used E-glass, polyethylene, and quartz fibers as FRC retainers as an alternative to traditional stainless steel retainers. A previous study demonstrated that these fibers were noncytotoxic, as evidenced by the absence of any adverse effects on fibroblasts from day 1 of the exposure until day 11 when the cells returned to their normal state.^24^ The exceptional compatibility of E-glass can be attributed to its chemical resistance in very acidic and mildly acidic conditions. It does not undergo any potentially harmful reactions, leaching, or release of substances that could be toxic to cells.^27^

 Quartz fiber, which falls under silica-based fibers, exhibits low cytotoxicity because of its fibrous structure. Balos et al^32^ found that the nanocomposite, which consisted of a matrix of silica-PMMA resin containing nanoparticles, exhibited cytotoxicity because of an increase in nanoparticle concentration. Specifically, the inclusion of nanoparticles inevitably alters the structure of the material, which may affect the biocompatibility of nanocomposites. This study used quartz fibers pre-impregnated with a unique methacrylic resin matrix containing crystalline silica, which may result in less cytotoxicity than the nanoparticle version. Conversely, Ikuno et al^33^ evaluated the cytotoxicity of undegraded and degraded polyethylene fibers. Undegraded FRCs showed low cytotoxicity in all polyethylene concentration ranges tested. However, the cytotoxicity rate increases when they are degraded because degradation reflects the peak height of the carboxyl groups, resulting in damage to the cell membrane. Furthermore, this finding suggested that increased carboxyl groups enhanced cytotoxicity. Notably, the results of this previous study demonstrated surface degradation dependence, which cannot be measured for simple surface-altered samples. Nevertheless, a degradation test was not performed in the present study; however, to maintain cell viability, the degradation of material used for FRCs must be minimized, and any safe fibers with low adverse effects, even after degradation, must be incorporated. This could be a potential future study to assess the degradation effect of natural silk fibers on cell viability.

 In addition, the flowable composite used for impregnation and production of FRCs has shown favorable biocompatibility.^24^ However, it must be applied correctly, as any leftover or unreacted monomers found to be the leading cause of cytotoxicity to fibroblasts can be minimized.^11^ Therefore, the concentration and type of resin monomers are noteworthy, particularly for restorations in direct contact with the gingival tissue.^34^

 This study was limited by its sole use of a single cell line. These cells exhibited diminished clinical simulation conditions. Moreover, the duration of exposure to the substance affected cell survival. This study specifically evaluated the effects of fibroblast cell line exposure for 24 hours. Nevertheless, this investigation adds to the initial findings concerning cytotoxicity. Future research should explore extended exposure to different cell lines or primary cells, compare direct and indirect contact methods, and incorporate more realistic clinical simulation circumstances.

## Conclusion

 The results of this study indicated that both natural silk and synthetic fibers did not have any harmful effects on fibroblast cell lines. Furthermore, the cytotoxicity of the composite resin used for impregnation must be considered. Cell viability of natural silk FRCs, specifically *B. mori* and *S. ricini*, was determined to be comparable with other synthetic FRCs currently on the market. Despite restrictions, the natural silk fibers from *B. mori* and *S. ricini* can be used as reinforcements in dental composites.

## Competing Interests

 The authors declare no conflicts of interest.

## Ethical Approval

 This study was approved by the Ethics and Advocacy Commission of the Faculty of Dentistry, Universitas Gadjah Mada, under ethical clearance number 73/UN1/KEP/FKG-RSGM/EC/2023.

## Funding

 Funding was earned from a community service grant from the Faculty of Dentistry, Universitas Gadjah Mada (Grant number: 3843/UN1/FKG/Set. KG1/LT/2023).
